# Anti-HSV Activity of Metallic Nanoparticles Functionalized with Sulfonates vs. Polyphenols

**DOI:** 10.3390/ijms232113104

**Published:** 2022-10-28

**Authors:** Emilia Tomaszewska, Katarzyna Ranoszek-Soliwoda, Katarzyna Bednarczyk, Agnieszka Lech, Martyna Janicka, Marcin Chodkowski, Maciej Psarski, Grzegorz Celichowski, Malgorzata Krzyzowska, Jarosław Grobelny

**Affiliations:** 1 Department of Materials Technology and Chemistry, Faculty of Chemistry, University of Lodz, Pomorska 163 St., 90-236 Lodz, Poland; 2 Laboratory of Nanobiology and Biomaterials, Military Institute of Hygiene and Epidemiology, Kozielska 4 St., 01-063 Warsaw, Poland; 3 Division of Microbiology, Department of Preclinical Sciences, Institute of Veterinary Medicine, Warsaw University of Life Sciences, 02-786 Warsaw, Poland

**Keywords:** antiviral nanoparticles, natural-origin antivirals, tannic acid, heparin-mimic ligands

## Abstract

Metallic nanoparticles exhibit broad-spectrum activity against bacteria, fungi, and viruses. The antiviral activity of nanoparticles results from the multivalent interactions of nanoparticles with viral surface components, which result from the nanometer size of the material and the presence of functional compounds adsorbed on the nanomaterial surface. A critical step in the virus infection process is docking and entry of the virus into the host cell. This stage of the infection can be influenced by functional nanomaterials that exhibit high affinity to the virus surface and hence can disrupt the infection process. The affinity of the virus to the nanomaterial surface can be tuned by the specific surface functionalization of the nanomaterial. The main purpose of this work was to determine the influence of the ligand type present on nanomaterial on the antiviral properties against herpes simplex virus type 1 and 2. We investigated the metallic nanoparticles (gold and silver) with different sizes (5 nm and 30 nm), coated either with polyphenol (tannic acid) or sulfonates (ligands with terminated sulfonate groups). We found that the antiviral activity of nano-conjugates depends significantly on the ligand type present on the nanoparticle surface.

## 1. Introduction

The fight against viral diseases is difficult, mainly because the virus is neither alive nor dead. Although we know much more about the structure of different viruses, which may seem relatively uncomplicated because it contains a nuclear capsid that consists of short incomplete sections of genetic material (DNA or RNA), the fight against viruses is very complicated. The mechanism of viruses’ action is still far away from complete understanding. Moreover, the structure of viruses is constantly changing. Therefore, it is challenging to develop effective methods of fighting against viruses.

One of the most common human pathogens nowadays is HSV, which is the name of two viruses: herpes simplex virus type 1 (HSV-1), associated with orofacial infections and encephalitis, and herpes simplex virus type 2 (HSV-2), causing genital infections. HSV-1 is the most familiar infectious disease that occurs worldwide and infects humans; it has no animal vector, so it spreads via person-to-person transmission and directly infects epithelial cells [1]. HSV-1 can migrate through a sensory nerve to the trigeminal cranial nerve ganglion and persists in a quiescent state in the nuclei of the neurons [2,3]. The symptoms of HSV infection are already well known to most people and include cold sores of the mouth and keratitis, but HSV may also cause life-threatening diseases in immunodeficient persons, including newborns, patients with human immunodeficiency virus (HIV), or patients undergoing immunosuppressive treatment [4]. The research shows that HVS-2 infection increases three to six-fold the risk of HIV infection [4]. Therefore, reducing HSV-2 infections can also reduce the spread of HIV infections. Moreover, recent findings prove that HSV infection may induce amyloid beta (Aβ) production and its deposition in the brain, which may lead to subtle cognitive disturbances [5] and increases the risk factor for Alzheimer’s disease [6,7]. Hence, developing effective antiviral preparations and an effective vaccine against HSV is critical.

There are several ways to fight against the HSV virus [8]. Most currently available antiviral therapies (acyclovir, idoxuridine, famciclovir, penciclovir, valacyclovir, ganciclovir, foscarnet, trifluridine) rely on the mechanism of inhibition of viral DNA polymerase [9]. Although those antiviral drugs can be highly effective, they require strict patient compliance and can have harsh side effects. Nanomaterials have the potential to overcome these obstacles and to offer opportunities for developing novel broad-spectrum nano-therapeutic platforms to combat viral infections. Nanomaterials are simple to functionalize and have comparable sizes to viruses, so there are many bio-interfacing opportunities between nanoparticle systems and viruses or virus-infected tissues. Moreover, nanomaterials’ chemistry and functionality can be tuned to bind to and neutralize different pathogens selectively.

Interesting nanomaterials for antiviral applications are nanoparticles. Several nanoparticles type have been reported to be good research candidates for NP-based antiviral therapies, e.g., CdTe quantum dots [10], Ag_2_S nanoclusters [11], AgNPs [12,13,14], AuNPs [15], silica nanoparticles [16,17], CuO NPs [18], ZrO_2_ NPs [19], ZnO [20], TiO_2_ [21] etc. The general mechanism of virucidal activity of nanoparticles is based on disruption of the structural integrity and inhibition of infectivity by direct interaction with the viral envelope or capsid proteins. However, the small size of nanoparticles and high surface area combined with the appropriate surface functionalization allow for tuning of the interaction with viruses in a multivalent manner. The surface modification with different ligands can enhance their affinity towards specific viruses and confers the multimodality of nanoparticles, which can further help to introduce nanoparticles’ additional functions, e.g., wound healing properties [22]. 

The entry of the virus into the host cell can be inhibited by blocking the virus-specific surface structures responsible for docking to the host cell [23]. The HSV entry begins with the attachment of viral glycoproteins to host cells. Heparan sulfate proteoglycans are responsible for adhesion of HSV and many other viruses, such as human immunodeficiency virus (HIV), dengue virus or respiratory syncytial virus (RSV) to the host cell membrane. Heparan sulfate, the constituent of heparan sulfate proteoglycans, is structurally similar to heparin. Hence, nanoparticles functionalized with heparin mimic ligands will naturally bind to viruses that target heparan sulfate proteoglycans. There are many reports showing that heparin-mimic nanoparticles exhibit antiviral properties. Ross and co-workers [16] functionalized silica nanoparticles with glycosaminoglycan mimetic ligand (sodium benzene sulfonate). They showed that conjugates act as HSV entry inhibitors, blocking viral attachment and penetration into the host cells. Sarid and co-workers [24] showed that AgNPs functionalized with sulphonate end groups ligands target the HSV-1 virus and lead to the blockage of viral entry into the cell and the prevention of subsequent infection. Baram-Pinto et al. [25] reported 3-mercapto-ethyl sulfonate-functionalized AuNPs as inhibitors of HSV-1 infection of Vero cells in vitro and reduced the virus spreading by blocking the virus entry to the cell. Cagno et al. [26] further investigated the sulfonate-functionalized AuNPs with different chain linkers to determine the impact of the ligand conformational flexibility on the antiviral activity of conjugates. The results obtained revealed that the modification of AuNPs with high-flexibility linkers (linkers with extended length -sodium 11-mercaptoundecane-1-sulfonate) increased antiviral activity in vitro in Vero cells, ex vivo in human epivaginal tissue cultures, and in vivo in mice compared to the high-flexibility linker (3-mercapto-ethyl sulfonate) [26].

Hydrolyzable tannins are the other group of compounds that can target viral glycoprotein-glycosaminoglycan interactions and inhibit HSV-1 infection [27]. Tannins are compounds of natural origin from the group of polyphenols that exhibit antiviral activity [28,29]. Several other bioactive compounds are known for their antiviral activity, e.g., flavonoids, terpenoids, polyphenolics, lignans, coumarins, chlorophyllins, alkaloids, furyl compounds, thiophenes, proteins, peptides, etc. [30,31,32]. The antiviral activity of these compounds has been attributed to various mechanisms of action, including antioxidant activities, inhibition of DNA and RNA synthesis, and inhibition of viral entry to the host cell [33]. The compounds of natural origin which exhibit antiviral properties can be conjugated with nanoparticles and used as antiviral preparations [14,34,35,36]. The low toxicity, low side effects, and reduced likelihood of promoting antiviral resistance make antiviral drugs based on natural compounds up-and-coming candidates to combat pathogens effectively [34]. Nabila et al. fabricated a curcumin-based nanoemulsion to improve curcumin’s solubility and cell uptake efficacy, and they obtained a significant virus inhibitory effect [37]. Lin et al. showed that hydrolyzable tannins (chebulagic acid and punicalagin) could target viral glycoprotein-glycosaminoglycan interactions, inhibit HSV-1 entry, and prevent binding penetration, cell-to-cell spread, and secondary infection [27]. Those results suggest that the hydrolyzable tannins may be useful as competitors for glycosaminoglycans in managing HSV infections. Our previous work showed that tannic acid attached to the surface of AgNPs could block viral entry [14]. We have also demonstrated that the ability of NPs to neutralize HSV-2 viruses is size-dependent, proving that nanoparticles with a size of about 30 nm exhibit significantly better reducing HSV-2 infectivity (both in vitro and in vivo) than 13 nm and 46 nm-sized nanoparticles [34]. Moreover, the induction of antiviral cytokines and chemokines production by tannic-acid modified AgNPs, together with decreased inflammatory reaction in vivo, suggests that their activity is not restricted to virus inactivation, but tannic-acid modified silver nanoparticles consist of a potential microbicide for herpesvirus infection in the mucosal tissues [34]. Both HSV-1 and HSV-2 possess similar structures of surface proteins. Hence, tannic acid-modified metallic nanoparticles can be used as a microbicide to treat both HSV-1 and HSV-2 types of infection. This makes them interesting materials, especially in the context of the development of anti-HSV vaccines. As the surface of HSV-2 is composed of glycoproteins with N- or O-linked sugars, it may readily interact with tannic acid-functionalized-NPs and help nanoparticles to interact with the virus envelope and inhibit the infection. Moreover, the nanoparticles may be a physical barrier in virus-host cell interaction by binding to the virus surface. These earlier reports provide a strong background for our studies and suggest that the tannins conjugated to metallic nanoparticles constitute an excellent goal for HSV therapeutics.

Based on the considerations above, we hypothesize that antiviral activity of functional nanoparticles depends on the ligands type present on their surface. Moreover, by adjusting the surface chemistry of nanoparticles, the properties of functional nanoparticles can be consciously designed and tuned to target a particular stage of viral infection. Hence, the goal of this study was to prepare and compare the inhibitory effect of two types of nano-conjugates known for their anti-HSV activity: sulfonate-conjugated metallic nanoparticles and tannic acid-conjugated metallic nanoparticles. For this purpose, we prepared gold and silver nanoparticles with the same metallic core size, functionalized either with sulfone or with tannic acid, and compared their antiviral activity against HSV-1 and 2 viruses by using in vitro Vero cell culture.

## 2. Results

### 2.1. Conjugates Characterization

**Figure 1:**
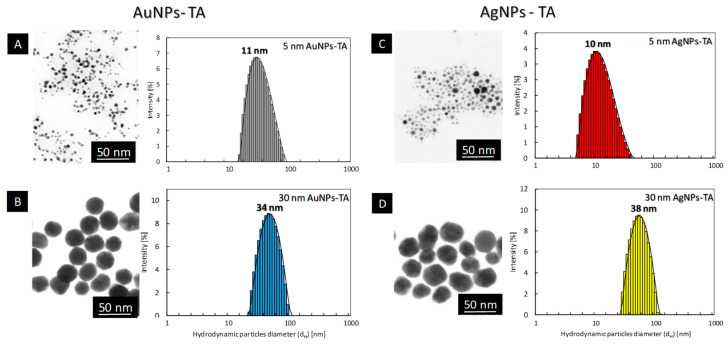
STEM images and DLS size distribution histograms of polyphenol-conjugated metallic nanoparticles: 5 nm AuNPs-TA (**A**); 30 nm AuNPs-TA (**B**); 5 nm AgNPs-TA (**C**); 30 nm AgNPs-TA (**D**).

**Figure 2:**
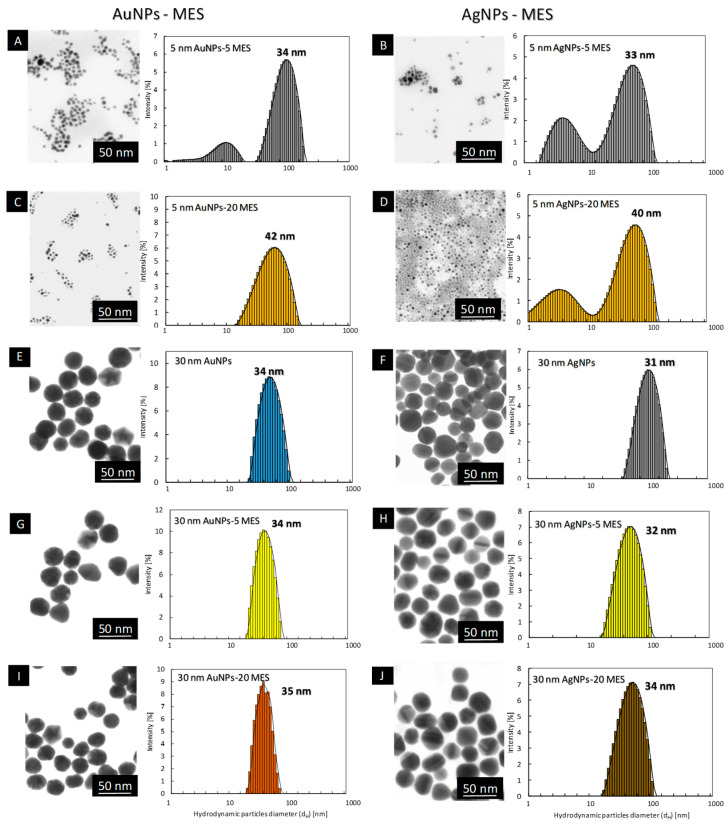
STEM images and DLS size distribution histograms of sulfonate-conjugated metallic nanoparticles: 5 nm AuNPs-5 MES (**A**); 5 nm AgNPs-5 MES (**B**); 5 nm AuNPs-20 MES (**C**); 5 nm AgNPs-20MES (**D**); 30 nm AuNPs (**E**); 30 nm AgNPs (**F**); 30 nm AuNPs-5 MES (**G**); 30 nm AgNPs-5 MES (**H**); 30 nm AuNPs-20 MES (**I**); 30 nm AgNPs-20MES (**J**).

**Figure 3:**
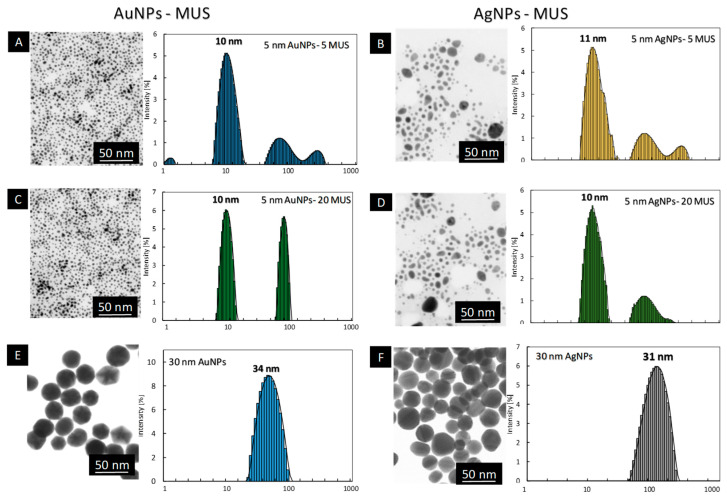

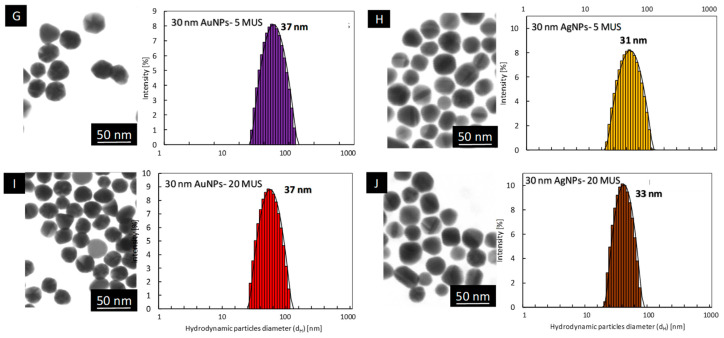
STEM images and DLS size distribution histograms of sulfonate-conjugated metallic nanoparticles: 5 nm AuNPs-5MUS (**A**); 5 nm AgNPs-5MUS (**B**); 5 nm AuNPs-20MUS (**C**); 5 nm AgNPs-20MUS (**D**); 30 nm AuNPs (**E**); 30 nm AgNPs (**F**); 30 nm AuNPs-5MUS (**G**); 30 nm AgNPs-5MUS (**H**); 30 nm AuNPs-20MUS (**I**); 30 nm AgNPs-20MUS (**J**).

To determine the effect of ligand type on the antiviral activity of the metallic NPs conjugates, gold and silver nanoparticles of the same shape and size were functionalized with tannic acid or sulfonic compounds, respectively. The precise characterization of nanoparticles functionalized either with tannic acid (Figure 1) or with MES-ligand (Figure 2), and MUS-ligand (Figure 3) was performed with HR-STEM and DLS to investigate the size, shape, size distribution, and colloidal stability of functionalized nanoparticles. In the case of TA-functionalized nanoparticles, the average particle size observed in HR-TEM was equal Au: 5 ± 2 nm, 31 ± 4 nm, Ag: 5 ± 2 nm, 27 ± 7 nm for colloids 5 nm AuNPs-TA (Figure 1A), 30 nm AuNPs-TA (Figure 1B), 5 nm AgNPs-TA (Figure 1C), 30 nm AgNPs-TA (Figure 1D), respectively. The shape of nanoparticles is spherical in all cases. DLS measurements showed the average hydrodynamic diameter of nanoparticles in each colloid equal: dH 5Au-TA = 11 ± 2 nm (Figure 1A); dH 30Au-TA = 34 ± 7 nm (Figure 1B); dH 5Ag-TA = 10 ± 2 nm (Figure 1C); dH 30Ag-TA = 38 ± 8 nm (Figure 1D).

The DLS measurements of MES-functionalized 5 nm AuNPs and AgNPs revealed the presence of peaks in the region corresponding to the value ~30–40 nm (Figure 2A–D). However, HR-TEM investigations of MES-functionalized nanoparticles revealed the presence of spherical objects in all cases, with the average particle size equal: 8.4 ± 3.8 nm for 5 nm AuNPs-5MES (Figure 2A), 9.1 ± 3.8 nm for 5 nm AgNPs-5MES (Figure 2B), 4.2 ± 2.0 for 5 nm AuNPS-20 MES (Figure 2C), 3.2 ± 0.7 nm for 5 nm AgNPs-20 MES Figure 2D). This indicates that MES-functionalized nanoparticles form conglomerates in the colloid, which were detected by the DLS technique as objects with a size of about ~30–40 nm. However, as presented in HR-STEM images, those conglomerates consist of single, separate particles without metallic connections between objects (Figure 2A–D). The HR-STEM measurements do not reveal any agglomerates or aggregates in any MES-functionalized colloid.

Nanoparticles with the size of about 30 nm functionalized with MES ligand were prepared by surface modification of citrate-stabilized nanoparticles (Figure 2E,F). Briefly, the physicochemical characteristic of those citrate-stabilized nanoparticles was as follow: the size of metallic core: dAuNPs = 31 ± 4 nm and dAgNPs 27 ± 7 nm, while the hydrodynamic diameter was equal: dH AuNPs = 34 ± 7 nm and dH AgNPs = 31 ± 8 nm, for AuNPs and AgNPs, respectively. Those nanoparticles were used for modification with MES ligand with the surface coverage equal 5 and 20 ligand molecules per 1 nm^2^ of nanoparticle surface. The physicochemical characterization of colloids after each functionalization is presented in Figure 2 G–J. The DLS results confirm the colloidal stability of nanoparticles after the functionalization process. The hydrodynamic diameter of MES-functionalized nanoparticles was equal: dH AuNPs-5MES = 34 ± 4 nm for 30 nm AuNPs-5 MES (Figure 2G), dH AgNPs-5MES = 32 ± 5 nm for 30 nm AgNPs-5 MES (Figure 2H), dH AuNPs20MES = 35 ± 6 nm for 30 nm AuNPs-20MES (Figure 2I), dH AgNPs-20MES = 34 ± 6 nm for 30 nm AgNPs-20MES (Figure 2J). The mean size of the metallic core of nanoparticles remains unchanged after the functionalization process and is equal dAuNPs = 31 ± 4 nm and dAgNPs = 27 ± 7 nm.

The physicochemical characterization of MUS-functionalized nanoparticles revealed the presence of spherical particles with the size of metallic core equal dAuNPs-MUS= 2.6 ± 1.0 nm for the syntheses 5 nm AuNPs-5MUS and 5 nm AuNPs-20MUS (Figure 3). The DLS measurements revealed the presence of intense peaks at 10 nm from single nanoparticles and around 100 nm from conglomerates (similar to the case of small nanoparticles synthesized with MES-ligand). Those conglomerates are formed in the solution and consist of single nanoparticles without the metallic connection between particles (STEM investigations do not revealed the presence of agglomerates or any larger objects). DLS measurements and the STEM investigations confirmed the stability of AuNPs functionalized with 5 and 20 MES molecules per 1 nm^2^ of NPs surface. The STEM images of AgNPs synthesized with 5 MUS molecules revealed the presence of spherical objects with the mean size of metallic core equal dAgNPs-MUS = 4.7 ± 1.6 nm. DLS measurements confirmed the colloidal stability of nanoparticles. The functionalization of 30 nm citrate AuNPs and AgNPs with MUS ligand allows for obtaining stable colloids, which was confirmed with DLS and STEM investigations (Figure 3G–J). The size of metallic core remains unchanged after MUS functionalization and is equal dAuNPs = 31 ± 4 nm and dAgNPs = 27 ± 7 nm. The DLS measurements revealed only small changes in the hydrodynamic size of nanoparticles after MUS modification, which is connected with the small size of the MUS molecule. The hydrodynamic size of MUS-functionalized 30 nm AuNPs increased from dH AuNPs = 34 nm measured for citrate AuNPs to dH AuNPs-5MUS = 37 nm for MUS functionalized AuNPs (both 5 and 20 MUS molecules per 1 nm^2^ of NPs surface). For 30 nm AgNPs the hydrodynamic size was equal dH AgNPs = 31 nm for citrate AgNPs and dH AgNPs-5MUS = 31 nm and dH AgNPs-20MUS = 33 nm for 5 and 30 MUS, respectively. The shape of nanoparticles functionalized with MUS-ligand is spherical in all cases.

### 2.2. Antiviral Activity of Conjugates

**Figure 4:**
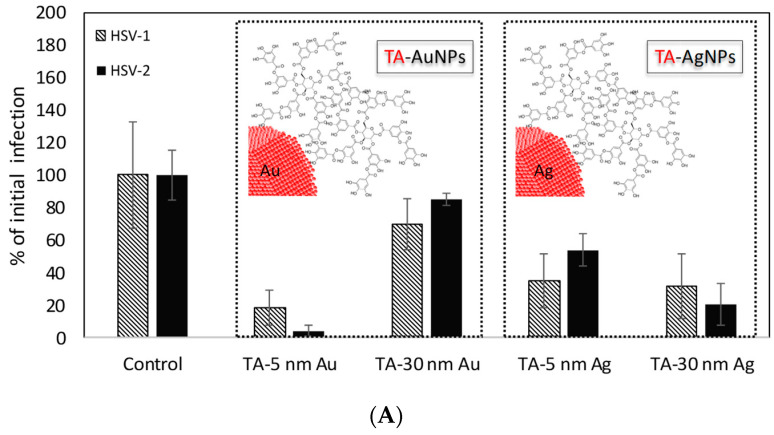

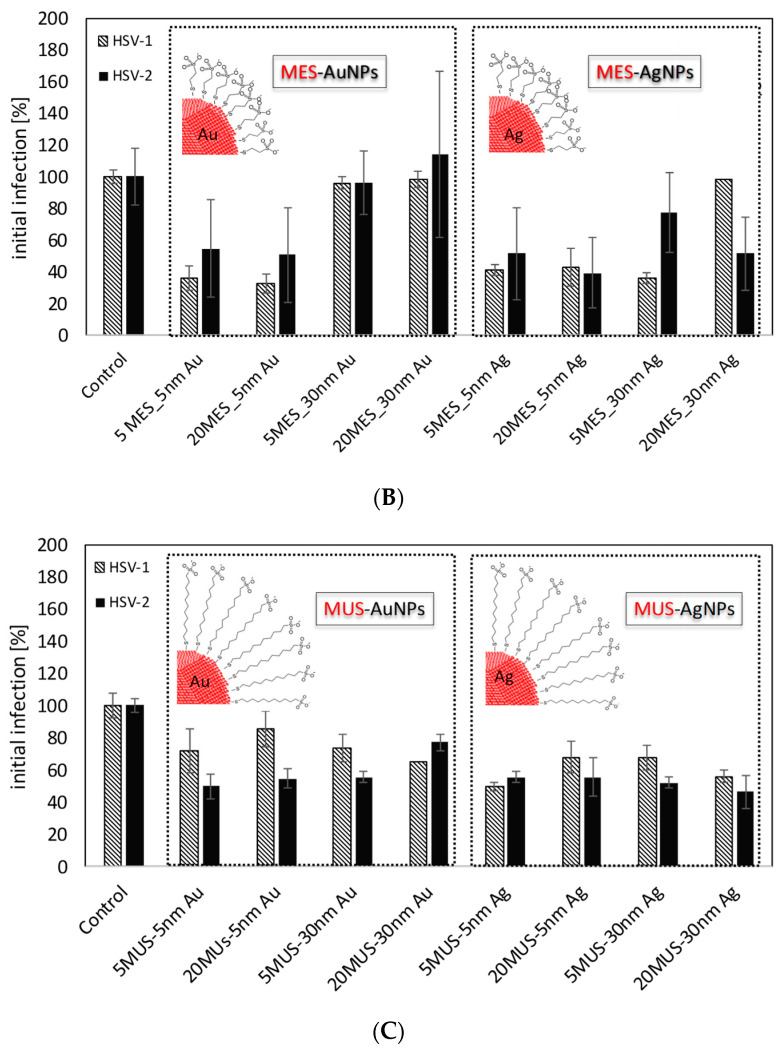
Inhibition of virus infection with the use of TA-conjugates (**A**), MES-conjugates (**B**), and MUS conjugates (**C**) of 5 and 30 nm AuNPs as well as 5 and 30 nm AgNPs. The efficiency of antiviral action was measured in plaque-forming units (PFU) and compared to untreated infected cultures. Results are expressed as % of untreated infection.

To compare the antiviral activity of conjugates, we tested the effect of different conjugates, sizes, and metal types upon HSV-1 or HSV-2 attachment and subsequent infection in Vero cells. Except for 30 nm AuNPs, all tannic acid conjugated Ag/AuNPs significantly prevented infection of Vero cells by HSV-1 or HSV-2 (*p* ≤ 0.04) (Figure 4A). For MES conjugates, only 5 nm AuNPs-5 MES/20MES, 5 nm AgNPs-5 MES/20MES, and 30 nm AgNPs-5 MES showed inhibiting activity against HSV-1 (Figure 4B). MES-modified 5 nm AgNPs-5 MES or 20 MES and 30 nm AgNPs-20 MES were effective against HSV-2 (Figure 4B). All MUS conjugates showed similar antiviral activity against HSV-2 (Figure 4C), while for HSV-1, only 5 nm AuNPs 20 MUS showed no inhibitory effect upon attachment and infection of Vero cells with HSV-1 (Figure 4C). The results of antiviral assays show that while the antiviral activity of tannic acid and MES conjugates depends on the size and metal (silver vs. gold) for tannic acid modification or size and ligand concentration (MES), the efficiency of MUS modification is related only with the chemical nature of this ligand, as all MUS conjugates show similar, approx. 50% reduction in HSV-1 or HSV-2 infection, irrespectively of the surface concentration of the ligand.

### 2.3. The Antiviral Mechanism of Functional Nanoparticles

**Figure 5:**
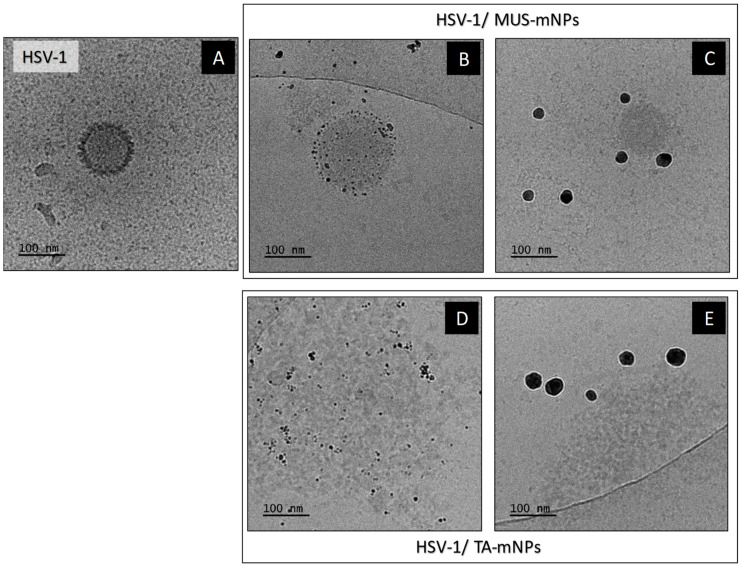
cryo-TEM images of unstained samples: HSV-1 virus (**A**), HSV-1 virus after incubation with: 5MUS_5nmAgNPs (**B**), 5MUS_30nmAgNPs (**C**) TA_5nmAgNPs (**D**), TA_30nmAgNPs (**E**) (samples imaged after about 30 s of incubation of virus with functional nanoparticles).

Cryo-TEM was used to visualize the interaction of HSV virus with functional nanoparticles and to identify and specify the mechanisms responsible for the action of selected functional nanoparticles. The cryo-TEM images of sulfonate-functionalized nanoparticles and tannic acid-functionalized nanoparticles are presented in Figure 5.

The Figure 5A presents the structure of untreated virus HSV-1. The analysis of the cryo-TEM images of sulfonate-NPs (Figure 5B,C) and TA-NPs (Figure 5D,E) revealed the differences in the number of nanoparticles in the vicinity of the virus for different nano-conjugates (Figure 5). In the case of sulfonate-functionalized nanoparticles, viruses immediately associate particles. The virus structure is covered with nanoparticles which can be easily seen in Figure 5B for 5MUS_5nmAgNPs. The high virus affinity to sulfonated nanoparticles can also be seen for nanoparticles with a size equal to 30 nm (5MUS_30nmAgNPs–Figure 5C). However, the analysis of the cryo-TEM images revealed that the sulfonated functional nanoparticles do not damage the envelope of viruses. The large number of nanoparticles adsorbed on the virus surface proves that they show a high affinity for its surface, and their mechanism of action is mainly based on the physical blocking of the interaction of the virus with host cell. In the case of tannic acid functional nanoparticles, the virus structure and the capsid are damaged (Figure 5D,E), eliciting the release of viral genetic materials leading to its destruction and virus inactivation. A lot of nanoparticles can also be visible in the vicinity of the damaged virus structures, indicating the virus’s high affinity to TA-mNPs and the high virucidal activity of functionalized nanoparticles. Those results suggest that the sulfonated-functional nanoparticles act rather virustatic, which is in agreement with Stellacci and co-workers’ [26] results, which reported heparan sulfate proteoglycans-mimicking materials effect as simply virustatic. At the same time, the tannic acid-functionalized nanoparticles exhibit a very strong virudical effect that causes irreversible viral deactivation by its degradation while maintaining low-toxic (Table S1 Supplementary Materials).

## 3. Discussion

The transmission of HSV virus occurs mainly by direct contact. The HSV infection disease begins with the adhesion of viral glycoproteins to the host cell membrane. The virus attaches by gB and/or gC envelope glycoproteins to heparan sulfate proteoglycans (HSPG) on the host cell surface [38]. This process opens a cascade of subsequent events, finally leading to virus fusion through the cell membrane. Heparan sulfate is present on the surface of almost all cell types as HSPG. Additionally, the highly sulfated heparin sulfate possesses negative charges that interact with the positively charged viral glycoproteins [39]. These interactions are favored, therefore heparin-mimic functional nanoparticles compete with cell docking targets, and hence, may prevent further entry and infection of the host cells. This leads to the inhibitory effect of sulfonated-NPs in HSV infections. Our results are in agreement with the results presented by Ross and co-workers [16], who showed that sodium benzene sulfonate-functionalized silica nanoparticles act as entry inhibitors and block viral attachment and penetration into the host cells. The results presented by Cagno et al. [26] indicate that the hydrocarbon chain length in the case of sulfone ligands also plays an essential role in the possibility of adsorption to the virus surface. The authors proved that the length of the hydrocarbon linker chain determines the ligand conformational flexibility and hence increases functional nanoparticle antiviral activity. We also observed that the efficiency of MUS functionalization is related only to the chemical nature of this ligand, as all MUS conjugates show similar, approx. 50% reduction in HSV-1 or HSV-2 infection. The tannic acid-functionalized nanoparticles exhibit higher virudical activity against both HSV-1 and -2 than sulfonate. This indicates various antiviral mechanisms of sulfonate- and tannin acid–modified nanoparticles. TA-functionalized nanoparticles induce damage to a fraction of the HSV viruses that is significantly higher compared to this observed for sulfonated-mNPs. The analysis of the cryo-TEM images showed that the action of TA-mNPs is very rapid and leads to the destruction of the virus. The virus structure is disrupted, the capsid material is released, and the viral replication machinery is stopped; consequently, no further infection of the cells is possible. Additionally, in our previous work, we have presented the additional adjuvant-like activity of TA-AgNPs, most probably induced by nanoparticles themselves [14,38,40]. Moreover, we also showed that TA-AgNPs overcome the inhibition of DCs maturation by live or inactivated HSV-2, typically observed in this infection model, which indicates that TA-functional AgNPs can be good activators of the immune response [14,40].

## 4. Materials and Methods

### 4.1. Chemicals

Gold (III) chloride hydrate (Sigma-Aldrich, ≥49%), tannic acid (C_76_H_52_O_46_, Sigma-Aldrich), sodium citrate (C_6_H_5_Na_3_O_7_∗2H_2_O) purity 99.0%, Sigma-Aldrich), silver nitrate (AgNO_3_, purity 99.999%, Sigma-Aldrich, St. Louis, MO, USA), sodium 2-mercaptoethanesulfonate (MES, C₂H₅NaO₃S₂, Sigma Aldrich), sodium 11-mercaptoundecane-1-sulfonate (MUS, C_11_H_23_NaO_3_S_2_, Prochimia, Poland), sodium borohydride (NaBH_4_, purity ≥96%, Sigma-Aldrich) were used as received. For all preparation of aqueous colloids, deionized water obtained from the Deionizer Millipore Simplicity UV system (specific resistivity of water was 18.2 MΩ·cm) was used.

### 4.2. Synthesis of Nanoparticles

#### 4.2.1. Polyphenol-Conjugated Metallic Nanoparticles

Aqueous colloids of gold and silver nanoparticles with the sizes 5 nm and 30 nm functionalized with tannic acid were synthesized by the chemical reduction method.

##### 5 nm AuNPs-TA

The preparation procedure of AuNPs with a size of about 5 nm was as follows: chloroauric acid water solution (93.80 g, 1.84·10^−4^% wt.) was boiled and vigorously stirred under reflux. Next, to the boiling solution, a mixture of sodium citrate (4.48 g, 0,877% wt.) and tannic acid (1.73 g, 1% wt) was added to the solution, and the mixture color changed from yellow to dark red, indicating the formation of the nanoparticle. The mixture was stirred for 15 min and cooled to room temperature.

##### 30 nm AuNPs-TA

Tannic acid conjugated-gold nanoparticles with a size of about 30 nm were prepared by functionalization of citrate-AuNPs prepared using a seed growth-mediated method. Seeds for the synthesis procedure were prepared as follows: chloroauric acid water solution (94.25 g, 2.01 × 10^−4^% wt.) was warmed to the boiling point under reflux. Next, sodium citrate water solution (5.75 g, 0.877% wt.) was added to the solution. The reaction was continued for 15 min and next cooled down to room temperature. The final concentration of gold nanoparticles was 100 ppm. As prepared, spherical AuNPs with a mean size equal 13 nm were used as seeds for the synthesis of AuNPs with the size of 30 nm according to the following procedure: 6.1 g of seed solution, water (37.93 g), and sodium citrate (4%, 0.97 g) were warmed to the boiling point under reflux under vigorous stirring. Next, to the warmed solution, a chloroauric acid water solution (15 mL, c = 0.062%) was added through the capillary by a syringe pump (capillary diameter 20 mm, flow rate 10 mL/h). After adding all reagents, the mixture was heated for an additional 15 min and cooled down to room temperature. A prepared colloid with a metal concentration equal 100 ppm and a metallic core of about 30 nm was used to prepare tannic acid conjugated-AuNPs by introducing an aqueous solution of tannic acid (4.181 g, 4%) to 100 mL of nanoparticles colloid. The tannic acid concentration in the colloid was equal 0.0315 wt. % (315 ppm).

##### 5 nm AgNPs-TA

AgNPs with the size of a metallic core of about 5 nm were prepared as follow: to an aqueous solution of silver nitrate a mixture of sodium citrate (4.2 g, 4%) and tannic acid (0.6 g, 5%) was added, and after about 2 s a solution of sodium borohydride (0.7 g, 2%) was incorporated into the solution. After the addition of reductants, the mixture color changed to brown, indicating the AgNPs formation.

##### 30 nm AgNPs-TA

The synthesis of AgNPs with a size of about 30 nm was carried out according to the following procedure: a mixture of an aqueous solution of sodium citrate (4.2 g, 4%) and tannic acid (0.6 g, 5%) was added to the silver nitrate solution (95.2 g, 0.017%) warmed to the boiling point. Immediately after the reagent’s introduction, the solution’s color changed to brown, indicating the formation of silver nanoparticles. The solution was vigorously stirred under reflux for an additional 15 min and cooled down to room temperature. The concentration of nanoparticles in all colloids was 100 ppm. The final concentration of tannic acid in all syntheses was led to the same level, equal 0.0315 wt. % (315 ppm). In the case of syntheses carried out with a smaller amount of tannic acid, it was added in an appropriate amount after the synthesis process. In the case of syntheses carried out without tannic acid, it was added into the colloid after synthesis in an amount that finally reached the appropriate concentration equal in all cases 0.0315 wt. %.

#### 4.2.2. MES-Functionalized Nanoparticles

##### 5 nm AuNPs-MES

The 2.-mercaptoethanesulfonate-conjugated AuNPs with a size of about 5 nm were synthesized via chemical reduction method according to the following procedure: gold salt (HAuCl_4_, 0.136 wt.%, 6,345 g) was mixed with deionized water (41.350 g) and sodium 2-mercaptoethanesulfonate (MESNa, HSCH_2_CH_2_SO_3_Na; 0.1 wt.%, 0.423 g/0.1 wt.%, 1.695 g) in the flat bottom flask and mixed vigorously for 5 min on the magnetic stirrer in the room temperature. The amount of MESNa added to the solution has been calculated to match the coverage: 5 and 20 molecules per 1 nm^2^ of nanoparticle surface. This amount of modifier molecules used for NPs functionalization corresponds to incomplete surface coverage in the case of using 5 molecules per 1 nm^2^ of NP surface, and full surface coverage in the case of NPs functionalization with modifier molecules equal 20 per 1 nm^2^ of nanoparticle surface. Next, the first portion of sodium borohydride was incorporated into the solution (0.8 wt.%, 0.33 mL), and the solution was vigorously mixed. After 60 min of stirring, the second portion of sodium borohydride (0.8 wt.%, 0.726 mL) was added and the solution was mixed for an additional 24 h.

##### 30 nm AuNPs-MES

The 2.-mercaptoethanesulfonate-conjugated AuNPs with a size of about 30 nm were prepared by functionalizing citrate-AuNPs with 2-mercaptoethanesulfonate. The AuNPs preparation procedure is described in Section 4.2. Synthesis of nanoparticles/4.2.1. Polyphenol-conjugated metallic nanoparticles/30 nm AuNPs-TA. The functionalization process was carried out by the introduction of an aqueous solution of MESNa into the colloid at room temperature. The surface coverage of MESNa was equal to 5 and 20 molecules per 1 nm^2^ of AuNP with the size of 30 nm, which corresponds to incomplete and full surface coverage with modifier compound, respectively.

##### 5 nm AgNPs-MES

Similar procedure as in the case of 5 nm AuNPs-MES was carried out in the case of 2-mercaptoethanesulfonate-conjugated AgNPs: silver nitrate (AgNO_3_, 1 wt.%, 1.181 g), deionized water (g) and sodium 2-mercaptoethanesulfonate (MESNa, HSCH_2_CH_2_SO_3_Na; 1 wt.%, 0.116 g/1 wt.%, 0.463 g) were mixed in the flat bottom flask for 5 min in the room temperature. Next, sodium borohydride (0.8 wt.%, 1.314 g) was added, and the solution was mixed for 24 h. The amount of MESNa added to the solution has been calculated to match the coverage: 5 and 20 molecules per 1 nm^2^ of AgNP surface, which correspond to incomplete and full surface coverage with modifier compound, respectively. The final concentration of nanoparticles in AuNPs and AgNPs colloids was 100 ppm.

##### 30 nm AgNPs-MES

AgNPs with the mean size of metallic core equal to 30 nm were prepared according to two-step procedure. In the first step, the silver seeds were synthesized, which were further used to prepare AgNPs with a size equal to 30 nm. The synthesis procedure was as follows: sodium citrate (0.228 g) and deionized water (95 g) were mixed and heated at 70 °C for 15 min. Next, silver nitrate (1.7 mL, 1%) was added, and then after 2 s sodium borohydride (2 mL, 0.1%) was added with the syringe pump (syringe capacity 2 mL, diameter 8.00 mm, flow rate 55 mL·h^−1^). After the addition of all reagents, the hole mixture was additionally heated for 60 min at 70 °C, and then the mixture was cooled down to room temperature. As the synthesis procedure was carried out in an open system (without reflux) after the process, the amount of deionized water was added by weight so that the colloid mass was finally equal 100 g. The metal concentration was equal 100 ppm. The second step of the 30 nm AgNPs synthesis was as follows: sodium citrate (3 g, 1%) and deionized water (75 mL) were heated for 15 min under reflux. Next, an aqueous solution of silver nitrate was added to the reaction mixture with the syringe pump (2.6 mL, syringe 10 mL, diameter 10 mm, flow 20 mL/h), and silver seeds were added (10 g). The whole mixture was heated for 1 h under reflux. After this time, the second portion of sodium citrate was added (3 mL, 1%) and the second portion of silver nitrate with a syringe pump (2.6 mL, syringe 10 mL, diameter 10 mm, flow 20 mL/h), and the mixture was heated for another 60 min. After this time, the colloid was cooled to room temperature. The final AgNPs concentration was 355 ppm, and for subsequent experiments this colloid was diluted to 100 ppm. The functionalization process was carried out by the introduction of an aqueous solution of MESNa into the AgNPs colloid at room temperature. Briefly, to the 5 g of 30 nm AgNPs (c = 100 ppm), 0.04599 g of 0.05% MESNa was added to obtain the surface coverage equal 5 molecules per 1 nm^2^ of AgNP, and 0.4600 of 0.2% aqueous solution of MESNa to obtain the surface coverage equal 20 molecules per 1 nm^2^ of AgNP.

#### 4.2.3. MUS-Functionalized Nanoparticles

##### 5 nm AuNPs-MUS

AuNPs functionalized with 11-mercaptoundecane-1-sulfonate were prepared with a chemical reduction method according to the following procedure: chloroauric acid water solution (3.807 g, 0.136 % wt.) and water (25.179 g) were added into a flat bottom flask and mixed vigorously for 5 min at room temperature. Next, sodium borohydride (1.015 mL, 0.5%) was added, and the solution was mixed for an additional 1h. The final concentration of nanoparticles in AuNPs was equal 100 ppm. The 11-mercaptoundecane-1-sulfonate-conjugated AuNPs with the size of metallic core equal 5 nm and surface coverage 5 and 20 MUS molecules per 1 nm^2^ of nanoparticle surface were prepared by introduction of an aqueous solution of sodium 2-mercaptoethanesulfonate (0.14998 g, 0.05% for surface coverage 5 MUS; 0.14998 g, 0.2% for surface coverage 20 MUS) into the 5 nm AuNPs colloid (5 g).

##### 30 nm AuNPs-MUS

The 11-mercaptoundecane-1-sulfonate-conjugated AuNPs with a size of about 30 nm were prepared by functionalization of citrate-AuNPs with sodium 11-mercaptoundecane-1-sulfonate. The AuNPs preparation procedure has been described in Section 4.2. Synthesis of nanoparticles/4.2.1. Polyphenol-conjugated metallic nanoparticles/30 nm AuNPs-TA. The 11-mercaptoundecane-1-sulfonate-conjugated AuNPs with the size of metallic core equal 30 nm and surface coverage 5 and 20 MUS molecules were prepared by modification of nanoparticles with MUS. The functionalization process was carried out by the introduction of an aqueous solution of MUS (0.025 g, 0.05% for surface coverage 5 MUS; 0.2% for surface coverage 20 MUS) into the colloid (5 g) at room temperature.

##### 5 nm AgNPs-MUS

AgNPs functionalized with 11-mercaptoundecane-1-sulfonate were prepared with a chemical reduction method according to the following procedure: silver nitrate aqueous solution (0.630 g, 0.5%) was mixed with an 11-mercaptoundecane-1-sulfonate aqueous solution (0.5512 g, 1%) and deionized water (18.259 g). Next, an aqueous solution of sodium borohydride (0.56 mL, 0.5%) was added, and the solution color was changed to brown; following this, the solution was mixed for an additional 1 h. The final surface coverage of AgNPs was equal 5 MUS molecules per 1 nm^2^ of NP surface. A sample with a surface coverage equal 20 molecules per 1 nm^2^ of AgNPs was prepared by incorporation of an additional amount of aqueous MUS solution (0.0413 g, 1%) to the 5 g of 5 nm AgNPs-5 MUS colloid.

##### 30 nm AgNPs-MUS

The 30 nm AgNPs-MUS preparation procedure was the same as that described for 30 nm AuNPs–MES, but the modification of AgNPs was carried out by incubation with the MUS compound. Briefly, to the 5 g of 30 nm AgNPs (c = 100 ppm), 0.04599 g of 0.05% MUS was added to obtain the surface coverage equal 5 molecules per 1 nm^2^ of AgNP, and 0.04599 of 0.2% aqueous solution of MUS to obtain the surface coverage equal 20 molecules per 1 nm^2^ of AgNP.

### 4.3. Nanoparticles Characterization

The characterization of AuNPs and AgNPs conjugates was performed using: Dynamic Light Scattering (DLS, Litesizer 500, Particle Analyzer, Anton Paar), High-Resolution Scanning Electron Microscopy equipped with transmission detector STEM II (HR-STEM, NovaNanoSEM 450, FEI, Hillsboro, Oregon, USA). STEM samples were prepared by drop-casting an aqueous dispersion of the nanoparticles onto carbon-coated copper grids. The size distribution of the NPs was analyzed by measurement of at least 500 nanoparticles for each sample and the histogram for the size distribution of particles was prepared.

### 4.4. Toxicity of Conjugates

The toxicity of 10 ppm/mL silver or gold nanoparticles modified with tannic acid (TA); 2-mercaptoethanesulfonate (MES), or 11-mercaptoundecane-1-sulfonate (MUS) measured by the MTT assay (-(4, 5-dimethyl-2-thiazolyl)-2, 5-diphenyl-2H-tetrazolium bromide in Vero cell line after 24 h exposure (Table S1). The cellular viability was calculated as the percentage of viable cells relative to the negative control. Based on results from MTT assay, MNTC (maximum non-toxic concentrations) was calculated as NPs concentration with ≤20% of non-viable cells, and these concentrations were used for in vitro antiviral studies (results presented in Supplementary Materials).

### 4.5. Antiviral Tests In Vitro

HSV-1 (strain McKrae) and HSV-2 (strain 333) were grown and titrated in Vero cells (ATCC^®^ CCL-81, Rockville, MD, USA) and kept at −80 °C until use. Vero cells were maintained in Dulbecco’s modified EMEM (DMEM) supplemented with 10% fetal calf serum, 10 U/mL penicillin, and 100 μg/mL streptomycin (GIBCO). Both cell lines were maintained in standard conditions (37 °C, 5% CO_2_). To analyze how NPs can influence viral attachment, cells were pre-chilled at 4 °C for 15 min, then co-treated with NPs and HSV-2 for 1 h. After this time, virus and NPs were removed, cell monolayers were washed with ice-cold PBS and further incubated at 37 °C. At 24 h post-infection, virus titers were determined by plaque assay, as described previously [30]. Results were expressed as % of infection in control, untreated infected samples.

### 4.6. Cryogenic Transmission Electron Microscopy (Cryo-TEM) Measurements

Cryogenic Transmission Electron Microscopy (cryo-TEM) images were obtained using a Tecnai F20 X TWIN microscope (FEI Company, Hillsboro, Oregon, USA) equipped with field emission gun, operating at an acceleration voltage of 200 kV. Images were recorded on the Gatan Rio 16 CMOS 4k camera (Gatan Inc., Pleasanton, CA, USA) and processed with Gatan Microscopy Suite (GMS) software (Gatan Inc., Pleasanton, CA, USA). Specimen preparation was done by vitrification of the aqueous solutions on grids with holey carbon film (Quantifoil R 2/2; Quantifoil Micro Tools GmbH, Großlöbichau, Germany). Prior to use, the grids were activated for 15 s in oxygen plasma using a Femto plasma cleaner (Diener Electronic, Ebhausen, Germany). Samples were prepared by incubation of HSV-1 virus with functional nanoparticles for about 30 s and then a droplet (3 μL) of the suspension was placed to the grid, blotting with filter paper and immediate freezing in liquid ethane using a fully automated blotting device Vitrobot Mark IV (Thermo Fisher Scientific, Waltham, MA, USA). After preparation, the vitrified specimens were kept under liquid nitrogen until they were inserted into a cryo-TEM-holder Gatan 626 (Gatan Inc., Pleasanton, CA, USA) and analyzed in the TEM at −178 °C.

## 5. Conclusions

In this paper we proved that the antiviral properties of functional nanoparticles result from the following parameters: the nanoparticles material core, the size of the nanoparticles, and the chemical structure of the active organic compounds attached to the surface of the nanoparticles. The presented results show that the conjugation of silver/gold nanoparticles with tannic acid and other substances mimicking heparin sulphate proteoglycans can consist of an effective anti-HSV agent. The antiviral tests revealed that nanoparticles functionalized with natural origin compounds (tannic acid) are more effective in antiviral activity against HSV infections than heparin-mimic compounds (sulfonates). The analysis of the mechanism of functional nanoparticles action against HSV virus revealed that TA-functionalized nanoparticles act very rapidly and induce damage to virus structure that confirms its virudical action. This effect is significantly higher for TA-mNPs compared to this observed for sulfonated-mNPs that exhibit mainly virustatic effect because of the blockage of the virus adhesion to the host cell. This confirms that TA-functionalized nanoparticles of noble metals (silver and gold) are more effective than sulfonate-functionalized NPs, and hence, TA-mNPs can be considerate a very promising class of new antivirals.

## Data Availability

Not applicable.

## Supplementary Materials

The following supporting information can be downloaded at: https://www.mdpi.com/article/10.3390/ijms232113104/s1.
