# Supplementary material for: Anti-HSV Activity of Metallic Nanoparticles Functionalized with Sulfonates vs. Polyphenols
Source: Int J Mol Sci. 2022 Oct 28;23(21):13104. doi: 10.3390/ijms232113104 (PMC9657688; doi:10.3390/ijms232113104)
Supplement: Supplementary file 1 [file ijms-23-13104-s001.zip › ijms-1965772-supplementary.pdf]

Supplementary material

Antiviral Activity of Metallic Nanoparticles Functionalized with Sulfonates vs. Polyphenols

Emilia Tomaszewska[1], Katarzyna Ranoszek-Soliwoda[1], Katarzyna Bednarczyk[1], Agnieszka Lech[1], Martyna Janicka[2,3], Marcin Chodkowski[2], Maciej Psarski[1], Grzegorz Celichowski[1], Malgorzata Krzyzowska[2], Jarosław Grobelny[1*]

[1]Department of Materials Technology and Chemistry, Faculty of Chemistry, University of Lodz, Pomorska 163 St.,90-236 Lodz, Poland

[2]Laboratory of Nanobiology and Biomaterials, Military Institute of Hygiene and Epidemiology, Kozielska 4 St., 01-063 Warsaw, Poland

[3]Division of Microbiology, Department of Preclinical Sciences, Institute of Veterinary Medicine, Warsaw University of Life Sciences, 02-786 Warsaw

Corresponding author: jaroslaw.grobelny@chemia.uni.lodz.pl

Table S1. Toxicity of 10 ppm/ml silver or gold nanoparticles modified with tannic acid (TA); 2-mercaptoethanesulfonate (MES) or 11-mercaptoundecane-1-sulfonate (MUS) measured by the MTT assay (-(4, 5-Dimethyl-2-thiazolyl)-2, 5-diphenyl-2H-tetrazolium bromide in Vero cell line after 24 exposure. The cellular viability was calculated as the percentage of viable cells relative to the negative control. All experiments were performed in triplicate. * represents significant differences with $p \leq 0.05$, ** $p \leq 0.001$ in comparison to negative control – untreated cells (Student t test). On the basis of results from MTT assay, MNTC (maximum non-toxic concentrations) was calculated as NPs concentration with ≤20% of non-viable cells and these concentrations were used for in vitro antiviral studies.

	% viable cells ± SEM
negative control	100 ± 17
AuNPs 5 nm TA	79 ± 11
AuNPs 30 nm TA	87 ± 9
AgNPs 5 nm TA	68 ± 12 *
AgNPs30 nm TA	78 ± 11
AuNPs 5nm 5MES	91 ± 8
AuNPs 5nm 20MES	89 ± 2
AuNPs 30nm 5MES	106 ± 7
AuNPs 30nm 20 MES	91 ± 5
AgNPs 5nm 5MES	104 ± 2
AgNPs 5nm 20MES	79 ± 1
AgNPs 30nm 5MES	101 ± 3
AgNPs 30nm 20MES	76 ± 2*
AuNPs 5nm 5MUS	102 ± 2
AuNPs 5nm 20MUS	113 ± 2
AuNPs 30nm 5MUS	64 ± 21**
AuNPs 30nm 20MUS	78 ± 7
AgNPs 5nm 5MUS	107 ± 9
AgNPs 5nm 20MUS	103 ± 8
AgNPs 30nm 5MUS	112 ± 14
AgNPs 30nm 20MUS	71 ± 15*
